# It Helps to Know Where to Look: Visual Identification of Unintentionally Resected Parathyroid Glands is Improved When Inspection is Directed by Near Infrared Autofluorescence Imaging

**DOI:** 10.1007/s00268-021-06200-6

**Published:** 2021-07-01

**Authors:** Quan-Yang Duh, Claire E. Graves

**Affiliations:** 1 Department of Surgery, University of California, UCSF-Mount Zion, 1600 Divisadero Street, San Francisco, CA 94115 USA; 2 Division of Surgical Oncology, Department of Surgery, University of California, Davis, USA

Hypoparathyroidism is the most common complication after total thyroidectomy. Permanent hypoparathyroidism is associated with an increased risk of renal failure, cardiovascular disease, fractures, cataract, and overall mortality [1]. The risk of postsurgical hypoparathyroidism is lower when thyroidectomy is performed by high-volume experienced surgeons. Identifying parathyroid glands during operation also decreases this risk—the more parathyroid glands identified by the surgeon, the less likely the patient will develop hypoparathyroidism.

Parathyroid gland function can be lost by unintentional (and, at times, unavoidable) removal, or by interrupting their delicate vascular supply by thermal or mechanical injury. Because parathyroid glands can survive and regain function when autotransplanted, it is a common practice for surgeons to routinely examine the thyroidectomy specimen for any unintentionally removed parathyroid glands. If a parathyroid gland is found, it is autotransplanted into well-vascularized muscle—typically into the ipsilateral strap muscles or sternocleidomastoid. The autotransplanted parathyroid tissue will usually survive and secrete parathyroid hormone (PTH) after a few weeks. Such autotransplantation of parathyroid tissue can lower the risk of permanent hypoparathyroidism in patients after total thyroidectomy.

An experienced thyroid surgeon would usually search for any unintentionally removed parathyroid glands by visually inspecting the thyroidectomy specimen, paying particular attention to their typical anatomic locations (posterior to the mid-thyroid lobe for the upper gland and around the inferior pole and central neck nodal basin for the lower glands). Additional scrutiny is paid to the locations associated with any missing glands not seen in situ during thyroidectomy dissection. If a parathyroid gland is found on the thyroidectomy specimen, it is excised and confirmed by frozen section or by measuring aspirate for PTH, then autotransplanted.

Recently, both image-based and probe-based near infrared autofluorescence (NIRAF) devices have been developed to help surgeons identify parathyroid glands during cervical operations [2]. Most of the effort has been directed toward identifying and preserving the parathyroid glands during thyroid dissection, so that surgeons can protect their blood supply and leave the glands in situ. The image-based NIRAF device has been shown in one multicenter prospective study to lower the risk of transient postoperative hypoparathyroidism [3].

These NIRAF devices can also be used ex vivo to identify parathyroid glands that have been excised unintentionally during thyroidectomy, as described in the study by Bellier and colleagues [4]. In this study, a NIRAF imaging device (Fluobeam®) was used to examine 116 resected thyroid lobes from 70 consecutive patients. There were 130 parathyroid glands (56% of the 232 glands expected) seen by the surgeon and left in situ. Without using the device, 12 parathyroid glands in ten resected thyroid lobes were identified by the surgeons and were autotransplanted. After they had been cleared by visual inspection, the lobes were subsequently examined with the NIRAF imaging device; 24 auto-fluorescent spots were found in 24 resected lobes. These areas were not previously identified by the surgeons in the initial general visual inspection. Upon directed visual inspection, 13 of these spots were designated as likely parathyroid glands by the surgeon and 11 were designated non-parathyroid. These designations were all confirmed by pathologic exam. On final pathology, there were an additional 15 intrathyroidal parathyroid glands, deeper than 2 mm surrounded by thyroid tissues, in 13 resected lobes. Overall, of the 17% (N = 40) of parathyroid glands unintentionally removed in these 70 patients, about a third (N = 12) were identified by the surgeon on initial general visual inspection and autotransplanted, another third (N = 13) were identified only after additional focused inspection directed by NIRAF imaging, and the remaining third (N = 15) were intrathyroidal and could not be salvaged. The remaining 27% of expected parathyroid glands likely remained in situ but were not identified visually by the surgeons during the operation.

The study by Bellier and colleagues [4] shows that even in a high-volume center with experienced surgeons, identification of unintentionally removed parathyroid glands can be significantly improved when visual inspection of the thyroidectomy specimen is directed by NIRAF imaging. Notably, the findings of the NIRAF device should be carefully interpreted by the surgeon, as nearly half of autofluorescence spots found by the device did not ultimately correlate to parathyroid tissue. When used as a surgical adjunct in experienced hands, NIRAF imaging can potentially lead to improved salvage and autotransplantation of unintentionally removed parathyroid glands, with the goal of decreasing the risk of hypoparathyroidism.

It is important to note that although NIRAF imaging may improve the rate of parathyroid gland salvage for autotransplantation, parathyroid glands function better in situ than when autotransplanted [5]. Thyroid surgeons should always strive to leave the parathyroid glands in situ whenever possible, rather than liberally excising and autotransplanting. Image-based and probe-based NIRAF devices may be useful in the training of thyroid surgeons and may lead us to a better understanding of parathyroid anatomy [2]. More studies will be needed to confirm its efficacy and evaluate its cost-effectiveness on decreasing the complication of post-thyroidectomy hypoparathyroidism.
